# Tuning the viscoelastic properties of peptide coacervates by single amino acid mutations and salt kosmotropicity

**DOI:** 10.1038/s42004-023-01094-y

**Published:** 2024-01-04

**Authors:** Xi Wu, Yue Sun, Jing Yu, Ali Miserez

**Affiliations:** 1 Biological and Biomimetic Material Laboratory (BBML), Center for Sustainable Materials (SusMat), School of Materials Science and Engineering, Nanyang Technological University, Singapore, 637553 Singapore; 2 School of Materials Science and Engineering, Nanyang Technological University, Singapore, 637553 Singapore; 3 Institute for Digital Molecular Analytics and Science, Nanyang Technological University, Singapore, 637553 Singapore; 4 School of Biological Sciences, 60 Nanyang Drive, NTU, Singapore, 636921 Singapore

**Keywords:** Drug delivery, Peptides, Rheology, Self-assembly

## Abstract

Coacervation, or liquid-liquid phase separation (LLPS) of biomacromolecules, is increasingly recognized to play an important role both intracellularly and in the extracellular space. Central questions that remain to be addressed are the links between the material properties of coacervates (condensates) and both the primary and the secondary structures of their constitutive building blocks. Short LLPS-prone peptides, such as GY23 variants explored in this study, are ideal model systems to investigate these links because simple sequence modifications and the chemical environment strongly affect the viscoelastic properties of coacervates. Herein, a systematic investigation of the structure/property relationships of peptide coacervates was conducted using GY23 variants, combining biophysical characterization (plate rheology and surface force apparatus, SFA) with secondary structure investigations by infrared (IR) and circular dichroism (CD) spectroscopy. Mutating specific residues into either more hydrophobic or more hydrophilic residues strongly regulates the viscoelastic properties of GY23 coacervates. Furthermore, the ionic strength and kosmotropic characteristics (Hofmeister series) of the buffer in which LLPS is induced also significantly impact the properties of formed coacervates. Structural investigations by CD and IR indicate a direct correlation between variations in properties induced by endogenous (peptide sequence) or exogenous (ionic strength, kosmotropic characteristics, aging) factors and the β-sheet content within coacervates. These findings provide valuable insights to rationally design short peptide coacervates with programmable materials properties that are increasingly used in biomedical applications.

## Abstract (web summary)

Peptide-based coacervates display interesting properties for biomedical applications, however, the link between peptide structure and coacervate material properties remains unclear. Here, the authors report a direct correlation between the primary and secondary structures of the peptides and the viscoelastic properties of the coacervates.

## Introduction

Over the past decade, coacervation, also termed liquid-liquid phase separation (LLPS) has drawn growing attention due to its role across multiple length scales of the biological landscape, from the intracellular to the extracellular space. LLPS is involved in phenomena as diverse as membrane-less organelles^1,2^, amyloid formation^3,4^, biological adhesives^5–7^, or the origin of life^8–10^, to name just a few examples. Biomacromolecules and synthetic polymers-based coacervates have also received a strong research interest owing to their translational potential as protocells^11–13^, microreactors^14,15^, microencapsulation^16,17^, drug delivery vehicles^18–20^, and transfection agents for gene editing^21^. The material properties of coacervate microdroplets play a central role in all these applications. Thus, establishing the molecular structure/property relationships of biomolecular coacervates is anticipated to provide molecular-level design guidelines for coacervates with tailored properties. Short peptides with low complexity sequences are convenient model systems for such studies because the addition or deletion of short motifs—or even single amino acid mutations—significantly regulate their supramolecular structures and biophysical properties^22–26^, enabling to establish links between sequence, structure, and properties. In addition, peptide-based coacervates are relatively simple to synthesize and purify and are capable of recruiting and releasing a surprisingly large diversity of guest biomacromolecules^11,20,21,27^, making them attractive for biomedical applications.

In our laboratory, we have been focusing on peptides derived from the Humboldt squid beak^28,29^, a hard yet non-mineralized mouthpart made of chitin and proteins. The dominant proteins in the rostrum region of the squid beak are a family of histidine-rich beak proteins (HBPs) that have been demonstrated to exhibit pH-induced self-coacervation, a mechanism that has been proposed to play a central role during beak biofabrication^30–32^. HBPs are characterized by the presence of modular repeats at their C-terminus that have been shown to govern coacervation^23,31^. Designing a series of truncated peptides from HBP tandem repeats (HB*peps*), phase separation was found to be driven by the penta-repeat GHGLY^23^. Upon pH increase from an acidic environment, phase separation is nucleated by transient His-Tyr hydrogen bonding followed by droplet stabilization via π-π stacking of Tyr side-chains. Follow-up studies established that His and Tyr residues located at the extremities of HB*peps* play a prominent role in driving the self-assembly of a topological network within the microdroplets^24^. Among HB*peps*, GY23 (GHGLY GAGFA GHGLH GFA GHGLY) and its derivatives exhibit a rich phase behavior, forming both gel-like structures and coacervate microdroplets depending on sequence specificity, pH, ionic strength, and concentration^23^. GY23 is built from two GHGLY repeats that are essential to initiate phase separation, as well as linker regions made of either hydrophobic (GAGFA/GFA) or charged (GHGLH) blocks. GY23 peptides containing charged linkers predominantly form liquid-like coacervates, whereas those containing the hydrophobic motifs favor gel-like structures. These features make GY23 an ideal model peptide for investigating how amino-acid mutations and simple peptide motifs are linked to the coacervates’ material properties.

In this study, GY23 was chosen as a starting model peptide to study its biophysical and material properties in the condensed state, including rheological properties, viscosity and interfacial tension. A series of GY23 peptide variants was designed with increased or decreased hydrophobicity by mutating single amino acids, and systematically evaluated by plate rheology and surface force apparatus (SFA). Our data indicate that the materials properties of GY23 coacervates can be tailored by mutation of specific residue hydrophobicity as well as by varying external factors such as ionic strength and species. Combining these results with Fourier-Transform Infrared (FTIR) and Circular Dichroism (CD) spectroscopic characterizations indicate that the extent of β-sheets within the coacervate phase, which can be controlled by amino acid mutations and counterion type and concentration, plays a key role in regulating the material properties. In addition, GY23 coacervates exhibit aging that can also be attributed to increased β-sheet content over time. The findings of this study provide molecular-level insights into coacervates’ materials properties as well as simple guidelines to design peptide coacervates with tunable physico-chemical properties for biomedical applications, such as bioadhesives, microreactors and therapeutic delivery.

## Results and discussion

### Synthesis and phase separation of GY23 variants

To study the effect of individual amino acid on the phase behavior, various peptide variants were synthesized by mutating individual or multiple amino acid residues in the linker region of GY23. The sequences of synthesized GY23 variants are summarized in Table 1. As highlighted in bold and underlined, the residues in the hydrophobic blocks were mutated to either more hydrophilic amino acids, namely phenylalanine (Phe) to leucine (Leu, 9 F/L) or alanine (Ala, 9 F/A), or to more hydrophobic amino acids by mutating Ala to Phe (7 A/F; 7 A/F-10A/F; 7 A/F-10A/F-18A/F). The hydrophobicity of GY23 variants can be estimated using the online peptide calculator^33^ and is shown in Table 1. The calculator defines the average hydrophilicity (AH) index of Gly (glycine) as zero, with AH increasing with the hydrophilicity of amino acid residues. All GY23 variants showed negative AH values as they are considered hydrophobic. With a single Phe→Leu and Phe→Ala mutation, the AH index increased from −0.80 to −0.77 and −0.72, respectively, suggesting decreased hydrophobicity of GY23-9F/L and 9 F/A. In contrast, AH index of GY23 variants decreased as the number of Ala→Phe mutations increased.

**Table 1:** Sequence of GY23 variants.

Peptide name	Sequence	AH index
GY23	GHGLY-GAGFA-GHGLH-GFA-GHGLY	−0.80
GY23-9F/L	GHGLY-GAG**L**A-GHGLH-GFA-GHGLY	−0.77
GY23-9F/A	GHGLY-GAG**A**A-GHGLH-GFA-GHGLY	−0.72
GY23-7A/F	GHGLY-G**F**GFA-GHGLH-GFA-GHGLY	−0.89
GY23-7A/F-10A/F	GHGLY-G**F**GF**F**-GHGLH-GFA-GHGLY	−0.98
GY23-7A/F-10A/F-18A/F	GHGLY-G**F**GF**F**-GHGLH-GF**F**-GHGLY	−1.07

Amino-acid mutations are bolded and underlined. Average hydrophilicity (AH) index values were calculated using the online peptide calculator (https://www.bachem.com/knowledge-center/peptide-calculator/). Negative values indicate that the peptides are hydrophobic.

**Fig. 1:**
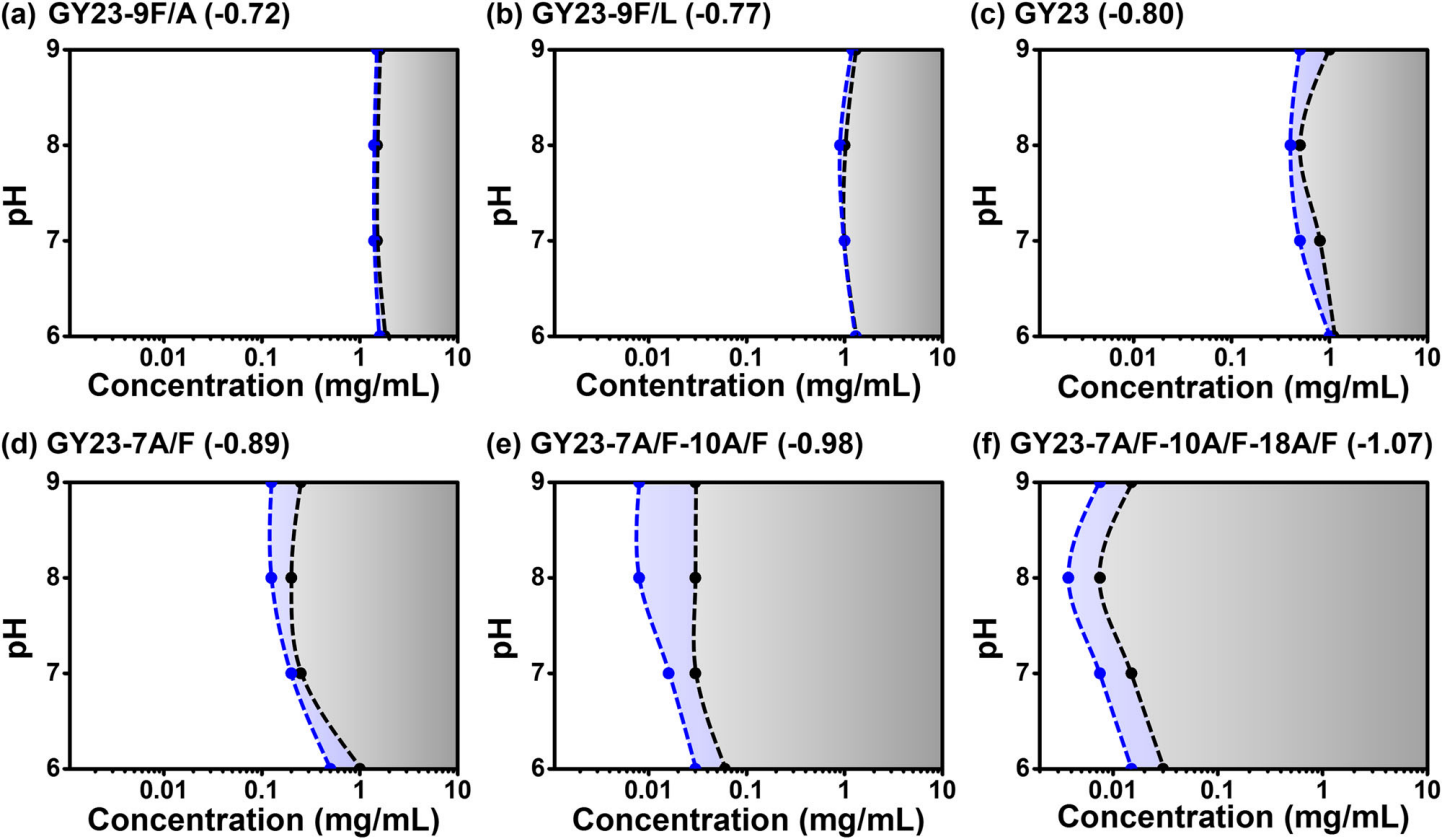
Critical phase separation concentrations of GY23 variants versus pH at 0.1 M and 1 M ionic strength fixed by NaCl obtained by optical microscopy. **a** GY23-9F/A. **b** GY23-9F/L. **c** GY23. **d** GY23-7A/F. **e** GY23-7A/F-10A/F. **f** GY23-7A/F-10A/F-18A/F. AH index of variants is indicated in the brackets. The two-phase regions are shadowed in gray (0.1 M) and blue (1 M). Coexistence of coacervates and aggregates are observed in the shaded area of (**e**) GY23-7A/F-10A/F and (**f**) GY23-7A/F-10A/F-18A/F.

The MALDI-TOF spectra used to verify the MW of the synthesized GY23 variants are shown in Supplementary Fig. 1. The synthesized GY23 variants were then studied for their coacervation ability by inducing coacervation in buffers with various pHs and ionic strengths. As the peptide hydrophobicity increased, the critical concentration to induce phase separation decreased, exhibiting a broader two-phase region as shown in Fig. 1. These results are consistent with previous studies, which indicated that hydrophobic interactions are essential for the self-coacervation of GY23 variants^23^. External factors such as pH and ionic strength also affect phase separation by charge screening and “salting-out” effects^34–36^. All peptides exhibited the lowest critical concentration for phase separation at pH 8.0, which is close to their isoelectric point of 8.01. At this pH, the net charge of GY23 variants reaches almost zero, thus minimizing electrostatic repulsions and promoting coacervation. Higher ionic strength induced a similar effect of shielding the charges from both of the N- and the C- termini and reducing the entropy loss of the counterion binding^37^, thereby shifting the critical concentration to slightly lower values.

**Fig. 2:**
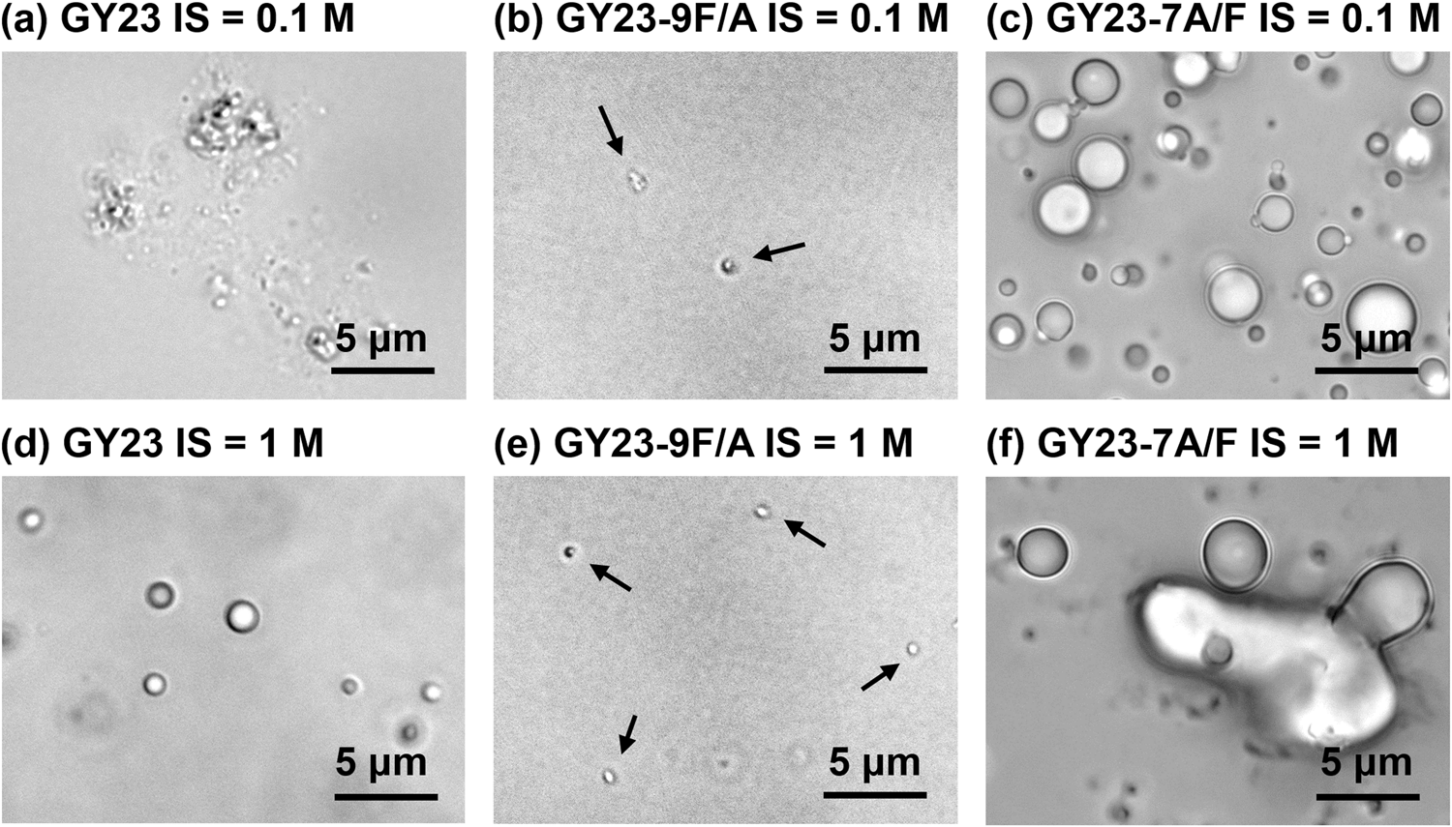
Representative optical micrographs of GY23 variant coacervates. **a**, **d** GY23, **b**, **e** GY23-9F/A, and **c**, **f** GY23-7A/F peptide coacervates (5 mg/mL) in pH 8.0 buffer with 0.1 M and 1 M of ionic strength fixed by NaCl. The black arrows in **b** and **d** highlight the few gel-like particles or coacervates formed by GY23-9F/A.

Representative micrographs of coacervates formed by GY23 variants are shown in Fig. 2. GY23 formed coacervates as regular and spherical microdroplets at high ionic strength but irregularly shaped gel-like structures at low salinity (Figs. 2a, d). This transition is likely caused by the charge screening effect at high ionic strength, whereby the charges of peptide molecules are neutralized by excess counterions, promoting the interaction among peptide molecules and the formation of the dense coacervate phase. To further support this hypothesis, we synthesized GY23 with blocked termini charges by acetylating the N-terminus and amidating the C-terminus (AA-GY23). As shown in Supplementary Fig. 2a, by removing the termini charges, AA-GY23 could form coacervate microdroplets at the lower ionic strength of 0.1 M. The increased ionic strength also promoted phase separation of AA-GY23 by the salting-out effect, facilitating the coalescence of coacervates and the formation of the denser phase (Supplementary Fig. 2b). Compared to GY23, a single Phe→Ala mutation (GY23-9F/A) was sufficient to affect the phase separation ability (compare Fig. 2d with 2e and Fig. 1a with Fig. 1c). In contrast, increasing the peptide hydrophobicity by mutating one Ala→Phe (GY23-7A/F) resulted in phase separation to occur at lower concentration (compare Fig. 1c with 1d) and this effect was further amplified with 2 (GY23-7A/F-10A/F) and 3 (GY23-7A/F-10A/F-18A/F) mutations, causing their precipitation (Supplementary Fig. 3).

### Viscoelastic properties of GY23 variant coacervates

**Fig. 3:**
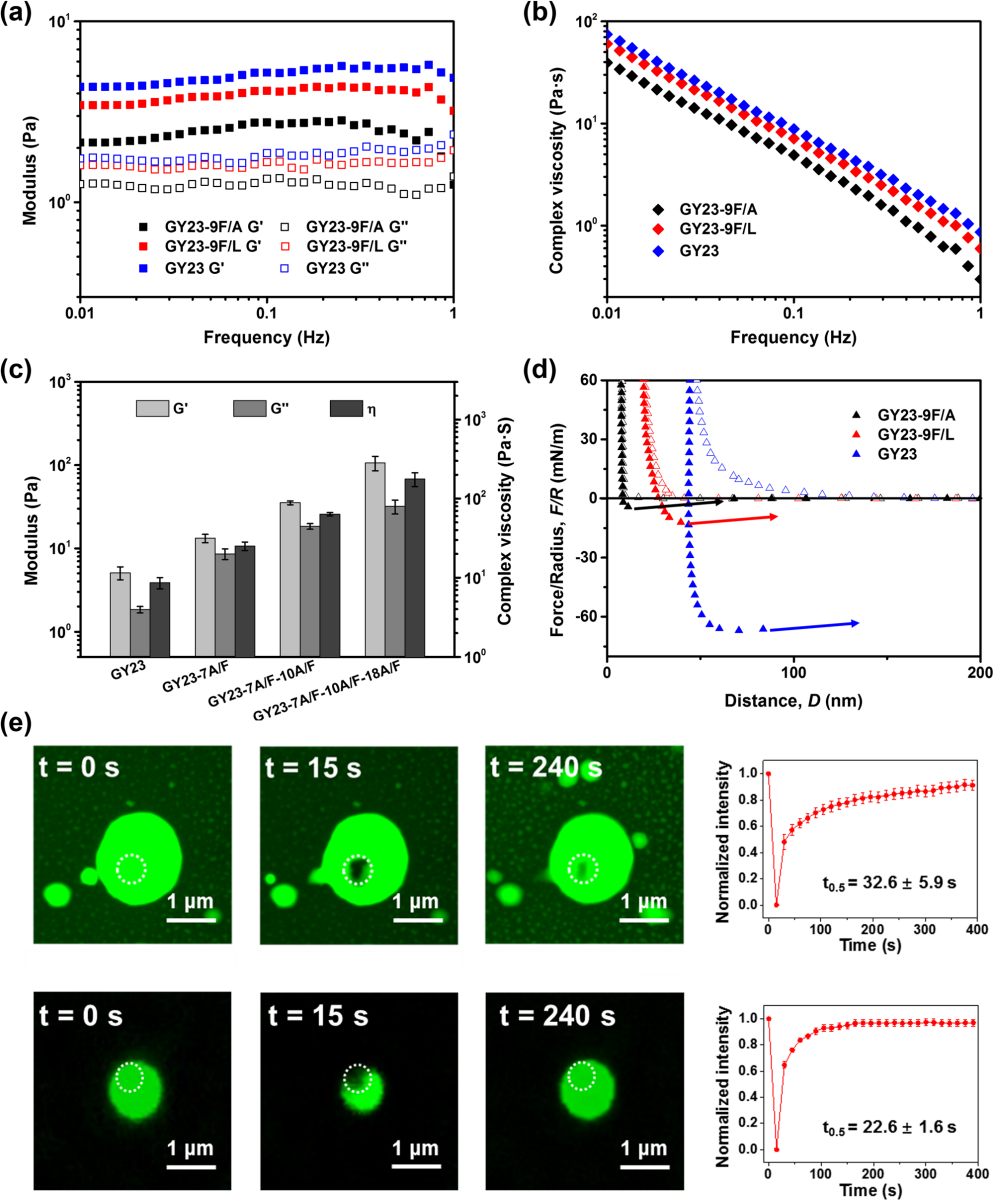
Properties of GY23 variant coacervates (5 mg/mL) formed at pH = 8.0 buffers with 1 M ionic strength fixed by NaCl. **a** Storage modulus (*G*’) and loss modulus (*G*”) of peptide coacervates. **b** Complex viscosity (*η*) of peptide coacervates. **c** *G*’, *G*” and *η* of coacervates formed by GY23 variants with A→F mutations at the sweep frequency of 0.1 Hz. Data are presented as the mean ± SD of *n* = 3 independent experiments. **d** SFA force-distance curves of GY23 peptide variant coacervates. All peptides formed coacervates at the test conditions, expect for GY23-7A/F-10A/F and 7 A/F-10A/F-18A/F, which formed precipitates due to stronger peptide-peptide interactions. **e** FRAP images and relative fluorescence intensities of GY23 (top) and GY23-9F/L coacervate microdroplets (bottom). Data presented as mean ± SD. *t*_0.5_ indicates the time required to achieve 50% recovery of maximum intensity. *t*_0.5_ of GY23-9F/L is significantly lower than GY23, two-sided Student’s *t* test, *P < 0.05.

To further explore the effect of single amino acid mutations on the viscoelastic and adhesive properties of peptide coacervates, rheological and SFA measurements were carried out. The storage and loss moduli of GY23 variants measured by plate rheology showed typical properties of a viscoelastic solid, with a ratio of *G*’/*G*” > 1 across the frequency range tested, and obvious shear thinning behavior as identified by the strong decay in complex viscosity at higher frequencies (Figs. 3a, b). With a single Phe→Leu mutation (GY23-9F/L), both the moduli and complex viscosity decreased *ca*. 20% compared to those of GY23, which can be attributed to reduced hydrophobic and aromatic interactions of Phe side-chains previously identified in GY23^23^. In contrast, coacervates or aggregates made of variants with one or more Ala→Phe mutations (i.e., GY23-7A/F, GY23-7A/F-10A/F and GY23-7A/F-10A/F-18A/F) exhibited 3-, 7- and 20-fold higher storage moduli and complex viscosity compared to GY23 at the frequency of 0.1 Hz, respectively (Fig. 3c and Supplementary Fig. 4). These results clearly demonstrate that the rheological properties of peptide coacervates can be readily tuned by simple adjustments of the primary sequence, such as Ala→Phe mutations.

The SFA is a nanomechanical technique that allows measuring intermolecular forces such as van der Waals, electrostatic, adhesion, and capillary forces^38–40^, and has been particularly successful in quantifying adhesive forces and interfacial energies of coacervates^41–43^. GY23, GY23-9F/L and GY23-9F/A were measured by SFA, but variants with one or more Ala→Phe mutations (such as GY23-7A/F and GY23-7A/F-10A/F) were prone to aggregation, which is not suitable for SFA measurements. Force-distance curves of three variants measured by SFA are shown in Fig. 3d. With the highest hydrophobicity, GY23 formed a coacervate layer with a thickness of 44 nm between two mica surfaces, shown as a “hard-wall” on the force-distance curves. Upon separation, a viscous jump-out was observed with a normalized adhesion force (*F*_*ad*_*/R*) of 66.8 mN/m, suggesting strong capillary adhesion of GY23 coacervate. This capillary adhesion measured by SFA is directly related to the interfacial energy $\gamma$ of the coacervate, which is an indication of the peptide-peptide interaction in the coacervate phase^44^. Replacing Phe→Leu led to smaller adhesion force and hard-wall thickness of the GY23-9F/L coacervate, resulting from the less aromatic and hydrophobic interactions of GY23-9F/L compared to GY23. Finally, the 9 F/A coacervate exhibited the smallest capillary adhesion *F*_*ad*_*/R* of 4.2 mN/m and hard-wall thickness (~8 nm), attributed to its decreased hydrophobicity and π-π stacking due to the Phe→Ala mutation. The SFA results are consistent with the rheological measurements, and corroborate that single amino acid mutations in short peptides significantly alter the viscoelastic properties of the resulting coacervates.

To further evaluate the molecular mobility in GY23 variant coacervates, fluorescence recovery after photobleaching (FRAP) experiments were conducted by using GY23 and GY23-9F/L coacervates containing 1% of the peptides KY24-FITC or KY24-9F/L-FITC (which have one extra Lys conjugated with FITC, see “Materials and Methods”). As shown in Fig. 3e, 9 F/L coacervates recovered 50% of fluorescence intensity after 22.6 s, which was significantly faster than the recovery rate of GY23 (32.6 s), suggesting that decreased hydrophobic interactions and π-π stacking enable higher mobility of peptide molecules within the coacervates. All these results indicate that the decrease of peptide interactions induced by Phe→Leu or Phe→Ala mutations disrupted the formation of dense structures in the resulting coacervates, further affecting their viscoelastic properties, which is consistent with a previous study showing that the addition of aromatic residues with spacing in the sequence favors phase separation^45^.

### Secondary structure characterization of GY23 variant coacervates

**Fig. 4:**
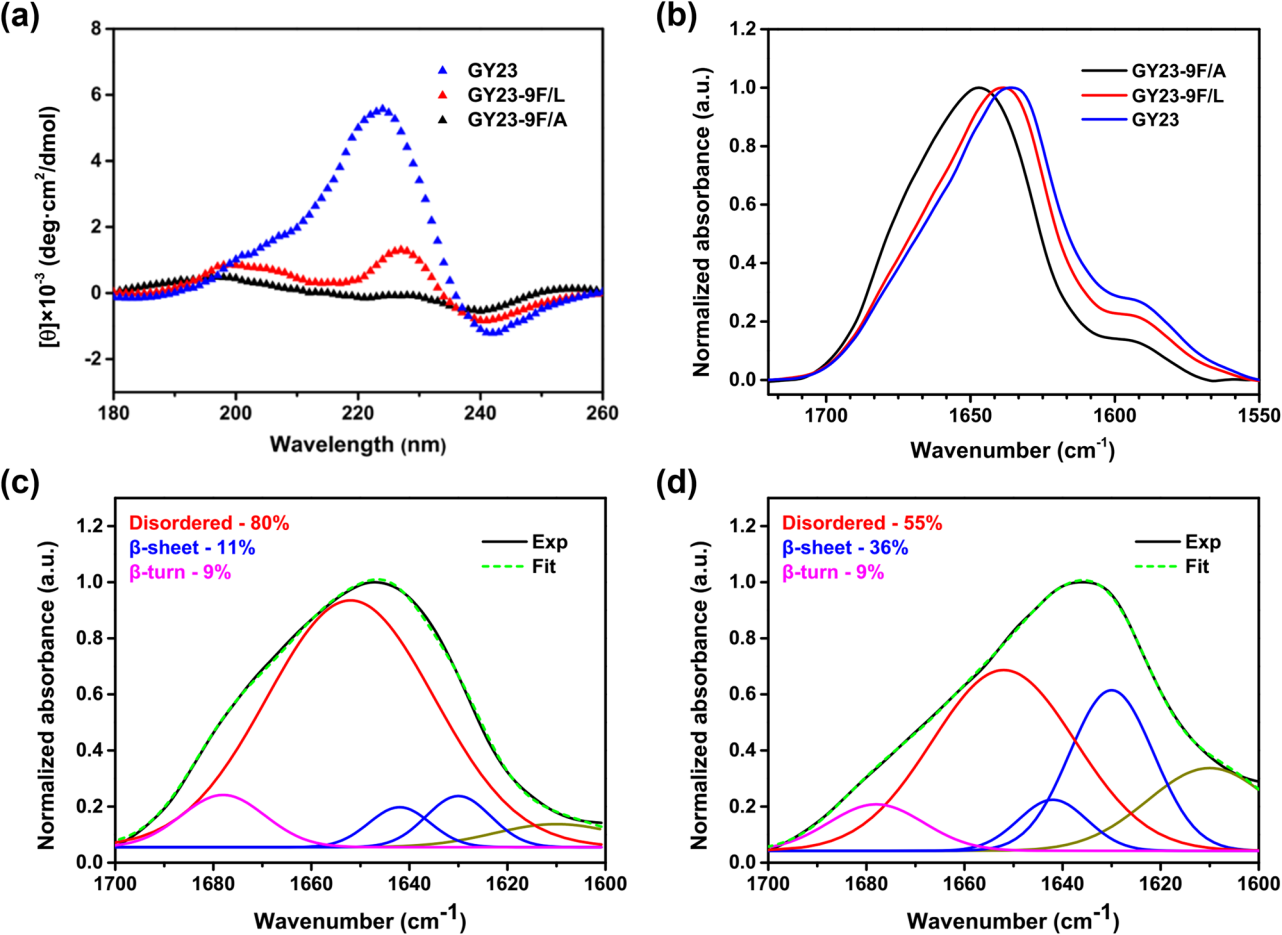
The secondary structure investigation of GY23 variant coacervates. **a** CD spectra of peptide variant coacervates. **b** FTIR spectra of GY23 peptide variant coacervates. **c**, **d** Deconvolution of the Amide I peak of GY23-9F/A (**c**) and GY23 (**d**) coacervates used to semi-quantitatively evaluate the relative ratios of secondary structures. All peptides formed coacervates at the test conditions.

Having demonstrated that the viscoelastic and adhesive properties of GY23 variant coacervates could be tuned by single residue mutations, we next aimed to assess how these variations may be linked to changes in secondary structures of the underlying peptides, which was carried out using CD and FTIR spectroscopy. The CD spectra of GY23 and GY23-9F/L (shown in Fig. 4a) were dominated by bands with maxima at 225 and 228 nm, respectively, which can be attributed to π-π* transitions arising from aromatic interactions (also called the Cotton effect)^46,47^. Due to the Phe→Leu mutation, GY23-9F/L showed weaker aromatic pair interactions (Tyr/Phe or Phe/Phe) compared to GY23, resulting in a significantly lower intensity and a slight shift of the π-π* band from 225 to 228 nm. As the hydrophobicity further decreased due to the Phe→Ala mutation, this band was essentially absent in GY23-9F/A, indicating reduced aromatic coupling in this variant. As shown in previous studies, aromatic interactions such as Tyr/Tyr and Trp/Trp play essential roles in stabilizing β-sheets^47–49^. The β-sheets formed by L-amino acid peptides are normally parallel, which gives a negative signal around 216 nm. However, for proteins with residues such as Gly, Ala, and Leu, anti-parallel β-sheets are favored over parallel β-sheets, which show a positive Cotton effect instead^50^. Therefore, the CD studies of these GY23 variants suggest that increased hydrophobic and aromatic interactions arising from single residue mutations favor the formation of more ordered structures, and eventually regulate the materials properties of peptide coacervates.

The FTIR spectra of GY23 variant coacervates, shown in Fig. 4b, provide additional cues regarding secondary structure differences between the different GY23 variant coacervates. Whereas the amide I band of GY23-9F/A was centered at 1652 cm^−1^, which is mainly attributed to a disordered structure, this band shifted to lower wavenumbers (*ca*. 1635 cm^−1^) for GY23 and GY23-9F/L, indicative of the presence of β-sheets^46,51^. Accordingly, deconvoluting the amide I band indicated a mostly disordered conformation for GY23-9F/A (88%) with a minor β-sheet contribution (*ca*. 11%) (Fig. 4c). In contrast, the β-sheet content was significantly higher in GY23 at *ca*. 36% (Fig. 4d). Given the established role of β-sheets in enhancing the mechanical properties of protein- and peptide-based structures^52–55^, the higher storage modulus of GY23 compared to GY23-9F/A can be attributed to its higher β-sheet content, which is itself favored by the more hydrophobic nature of GY23.

### Effect of ionic strength and salt kosmotropicity on viscoelastic behavior

**Fig. 5:**
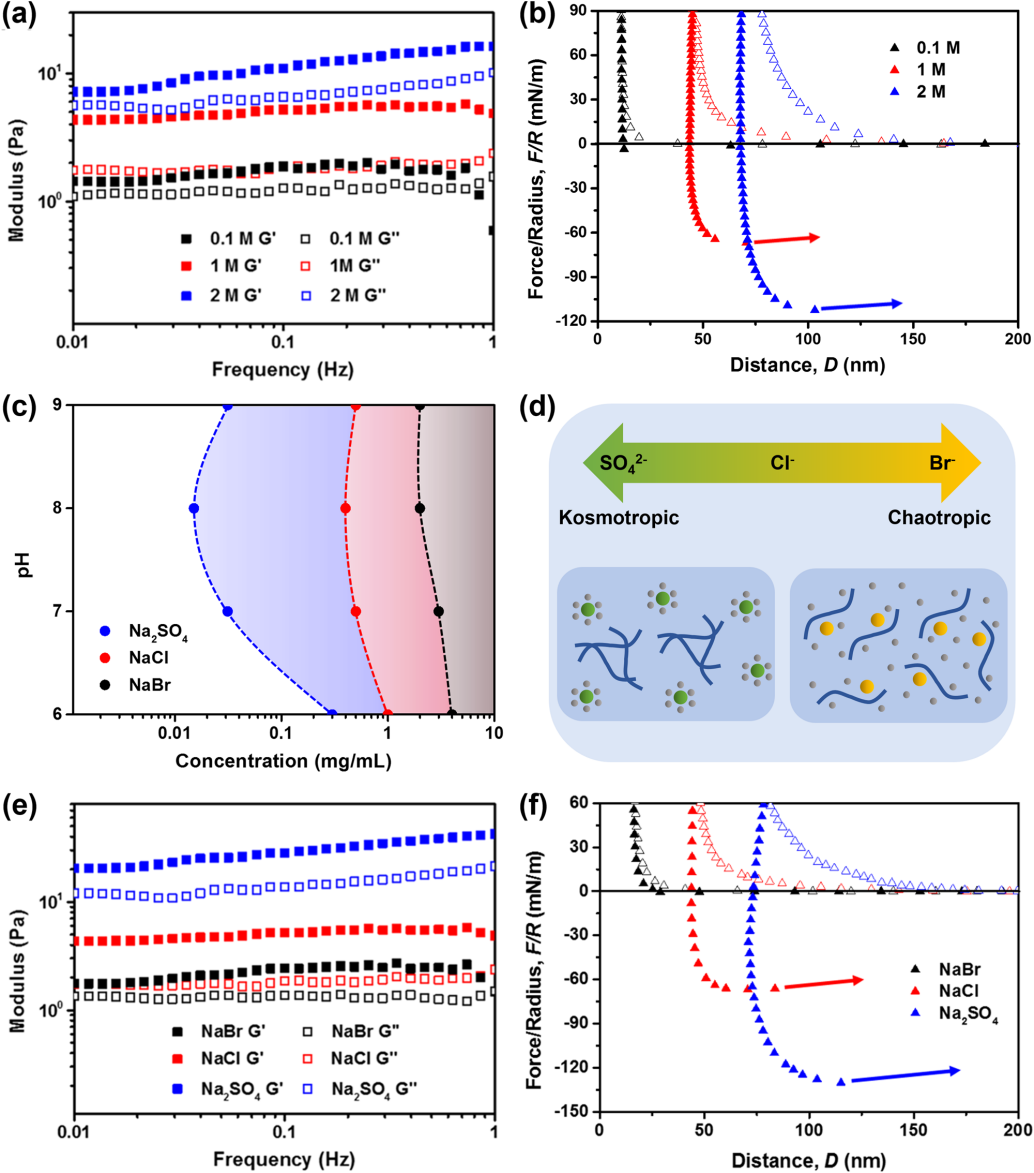
The effect of microenvironment on the viscoelastic and adhesion properties of GY23 coacervates (5 mg/mL). **a** Storage modulus (*G*’) and loss modulus (*G*”) of GY23 coacervates at various ionic strengths fixed by NaCl. **b** SFA force-distance profiles of GY23 coacervates at various ionic strengths fixed by NaCl. **c** Critical phase separation concentrations of GY23 (pH vs. peptide concentration) in 1 M ionic strength of various sodium salts obtained by optical microscopy. The two-phase regions are shadowed in black (NaBr), red (NaCl), and blue (Na_2_SO_4_). **d** Schematic illustration of the Hofmeister effect on GY23 peptide coacervates. Kosmotropic ions (green spheres), such as SO_4_^2−^ compete with peptide molecules (blue line) to interact with water molecules (gray spheres), facilitating the self-assembly of peptides into coacervates. On the other hand, chaotropic ions (yellow spheres) preferentially interact with peptides, thus delaying coacervation. **e** Storage modulus (*G*’) and loss modulus (*G*”) of GY23 coacervates formed in pH = 8.0 buffers with 1 M ionic strength of various sodium salts. **f** SFA force-distance profiles of GY23 coacervates formed in pH = 8.0 buffers with 1 M ionic strength of various sodium salts. All peptides form coacervates at the test condition.

To assess whether external variables such as ionic strength and ionic species also affect the material properties of peptide coacervates, we performed rheological and SFA measurements of GY23 at different NaCl concentrations and in different ions of the Hofmeister series. As the NaCl concentration varied from 0.1 to 2 M, *G*’ of GY23 coacervates increased by nearly 1 order of magnitude, from *ca*. 0.6 Pa to 10.2 Pa at the sweep frequency of 0.1 Hz (Fig. 5a). Additionally, the ionic strength strongly influenced the force-distance profiles during SFA measurements (Fig. 5b). At 0.1 M NaCl, no adhesive force was detected, which is not surprising given that GY23 could not form coacervate microdroplets at this salinity (Fig. 2a). However, benefiting from the charge screening and salting-out effects at 1 M ionic strength, GY23 formed a dense layer of coacervates and exhibited a strong adhesive force of *ca*. 70 mN/m, which further increased to almost 120 mN/m at 2 M NaCl. While these force values exceed those measured by SFA in mussel adhesive proteins (e.g., mussel foot protein 5, *F*_ad_/*R* ~ 65 mN/m)^56^ and other bioadhesives such as recombinant sucker ring teeth proteins (*F*_ad_/*R* ~ 70 mN/m)^57^, they are not directly related to the adhesive characteristic of the coacervates, but rather to the interfacial energy of the viscous film formed by confinement-induced coalescence of coacervates between the crossed cylinders. At increasing salt concentrations, intermolecular charge-charge repulsion decreases due to charge screening of His residues as well as C- and N-termini in peptides. Instead, attractive π-π stacking between Tyr residues is favored, which has been shown to accelerate the kinetics of phase separation^58^. Higher salinity also facilitates the dehydration of the peptide molecules^59^, promoting hydrophobic interactions which play essential roles in the phase separation of GY23. The present data show that this charge screening, as well as dehydration by the salting-out effect, not only affect the kinetics of phase separation but also results in more viscous coacervates due to enhanced intermolecular attraction within the coacervates.

Based on these findings, we surmised that the viscoelastic properties of GY23 coacervates could also be tuned by inducing phase separation in different salts of the Hofmeister series. Fig. 5c compares the critical phase separation concentrations of GY23 in NaCl as well as in the more kosmotropic sodium sulfate (Na_2_SO_4_) and the more chaotropic sodium bromide (NaBr). Compared to NaCl, coacervates prepared in Na_2_SO_4_ exhibited a broader two-phase region, for example enabling coacervation to occur at 0.015 mg/mL of peptide concentration at pH 8. By contrast, NaBr disrupted coacervation and allowed phase separation to occur only at higher peptide concentrations above 2 mg/mL at all pHs. These results are in line with the classical Hofmeister theory^60^. The kosmotropic SO_4_^2-^ ion competes with the peptide to interact with water molecules, thus favoring peptide-peptide interactions and consequently, phase separation (Fig. 5d). On the other hand, the chaotropic Br^−^ ions tends to interact with peptide molecules. Therefore, a higher peptide concentration is required to induce coacervation. Similar to the effect of ionic strength, different anionic species also strongly affected the viscoelastic properties. GY23 coacervates formed in 1 M NaBr showed around one magnitude lower storage moduli compared to those formed in Na_2_SO_4_ (Fig. 5e). Additionally, no adhesive forces were detected by SFA for GY23 in 1 M NaBr, whereas the adhesive force became increasingly prominent for more kosmotropic ions, reaching maximum values as high as 130 mN/m for coacervates prepared in Na_2_SO_4_ (Fig. 5f), again highlighting the more viscous nature of coacervates with strengthened peptide-peptide interactions.

Overall, the effect of buffer type (ionic strength and kosmotropicity) on phase separation of GY23 provides valuable guidelines to program the viscoelastic properties of peptide coacervates to meet specific targets. For example, recent evidence points out that uptake of peptide coacervates in mammalian cells is a mechano-sensitive mechanism^61^, with the implication that transfection efficiency depends on the viscoelastic moduli of the coacervates, similar to what is known with phagocytosis^62^. From that perspective, our data show that both internal variables (simple modifications of the peptide sequence), as well as external conditions (counterion type and concentration), can be modified to map a broad range of viscoelastic properties, which may enable to optimize the cell uptake of peptide coacervates for specific cell types.

### Ageing of GY23 coacervates

**Fig. 6:**
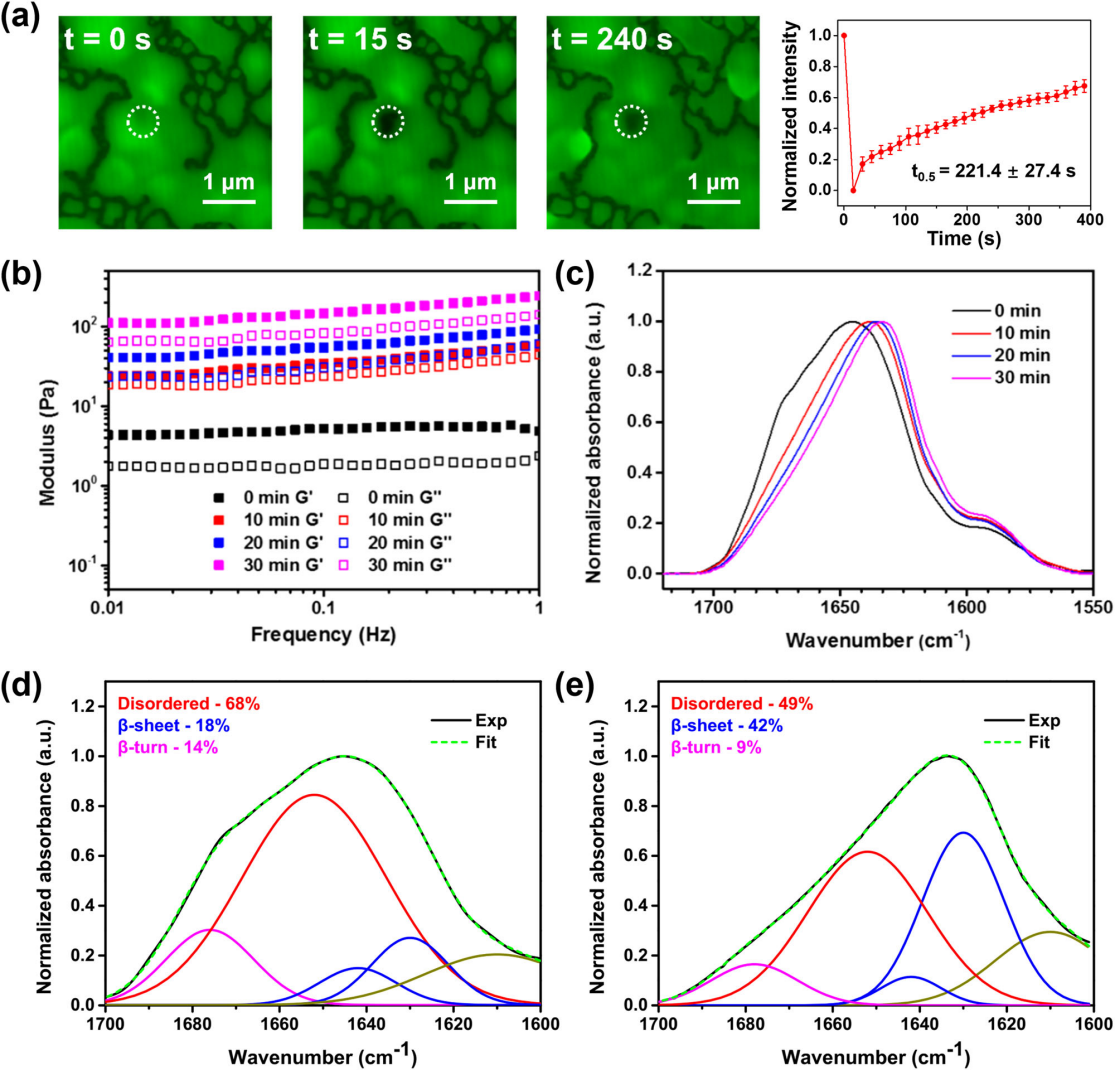
Aging phenomenon of GY23 coacervates. **a** FRAP images and relative fluorescence intensities of GY23 coacervates after 15 minutes incubation. Data presented as mean ± SD, *t*_0.5_ indicate the time required for 50% recovery to maximum intensity, which is significantly lower than freshly made GY23 coacervates in Fig. 3e, two-sided Student’s *t* test, *P < 0.001. **b** Storage modulus (*G*’) and loss modulus (*G*”) of GY23 coacervates incubated at room temperature for various time periods. **c** FTIR spectra of GY23 coacervates after incubation at room temperature for various time periods. **d**, **e** Curve fitting of the amide I peak of GY23 coacervates incubated at room temperature for 0 minutes (**a**) and 30 minutes (**b**), showing the shift towards b-sheet structures over time. All peptides form coacervates at the test conditions.

Since most coacervates exist in a metastable state of their constitutive proteins and peptides^63^, they may exhibit aging over time as the peptide transition towards the equilibrium state. In the case of GY23 coacervates, we also observed a fast aging process. After 15 minutes of incubation at room temperature, the GY23 coacervate droplets quickly coalesced, forming a viscous layer characterized by an obvious decrease in recovery rate compared to freshly made GY23 coacervates (Figs. 3e and 6a). Rheological measurements also confirmed the enhancement in material properties during the aging process, with the storage modulus of GY23 coacervates increasing by over one order magnitude, reaching *ca*. 10^2^ Pa after 30 minutes (Fig. 6b). To evaluate structural changes during aging, FTIR spectra of G23 coacervates at various time points were collected. The amide I peak gradually shifted to lower wavenumbers (Fig. 6c), indicating a structural transition towards β-sheets within the coacervates. Deconvolution of the amide I peak showed that the β-sheet content increased from 18% to 42% in 30 minutes after phase separation (Fig. 6d, e), suggesting that the aging phenomenon results from the formation of more ordered β-sheet structures within the coacervates. Promising applications of coacervates are in the area of bioadhesives^64–66^, and these data suggest that freshly made coacervates that readily coat and spread onto a surface due to their low interfacial tension would cure by aging over time with increased adhesion.

## Conclusion

In summary, this study shows that the viscoelastic properties of GY23 peptide coacervates can be tuned by systematically adjusting the hydrophobicity of specific residues in the peptide sequence. Mutating one single Ala to the more hydrophobic Phe facilitates phase separation, an effect that is amplified with 2 and 3 Ala→Phe mutations. Inversely, mutating Phe to the more hydrophilic Leu or Ala delays phase separation. These mutations also affect the moduli of the coacervates and their adhesive properties, with more hydrophobic peptides exhibiting higher storage moduli and viscous adhesion, and these changes in properties are correlated to the β-sheet content within the coacervates. The link between viscoelastic properties and β-sheet content is also confirmed in aging studies of GY23 coacervates. In addition, the viscoelastic properties can also be tuned by adjusting the ionic strength and species in the phase-separation buffer. Conditions that favor intermolecular interactions between peptide chains (higher ionic strength and komotrophic ions) facilitate phase separation and results in microdroplets with higher viscoelastic moduli and viscous adhesive forces. The ability to produce simple peptide coacervates with programmable material properties has valuable translational implications, notably for intracellular therapeutic delivery, since the uptake of microdroplets may depend on their viscoelastic characteristics.

## Materials and methods

### Materials

The tyrosine pre-loaded resin and fluorenylmethoxycarbonyl (Fmoc)-protected amino acids used in peptide synthesis were purchased from GL Biochem. N,N′-diisopropylcarbodiimide (DIC) and trifluoroacetic acid (TFA) were purchased from Tokyo Chemical Industry (TCI). Acetic acid, α-cyano-4-hydroxycinnamic acid (CHCA), deuterium oxide (D_2_O), fluorescein isothiocyanate (FITC), piperidine, potassium fluoride (KF), triisopropylsilane (TIPS), sodium chloride (NaCl), sodium bromide (NaBr) and sodium sulfate (Na_2_SO_4_) were purchased from Sigma-Aldrich. Acetonitrile (ACN), dichloromethane and N,N-dimethylformamide were purchased from Thermo Fisher Scientific.

### Peptide synthesis and purification

The peptides used in this study were synthesized by solid-state peptide synthesis using an automated peptide synthesizer (Liberty Blue model, CEM) and DIC/Oxyma as coupling reagents. After the synthesis, the peptide was cleaved from the resin using a cocktail containing 95% of TFA, 2.5% of H_2_O, and 2.5% of TIPS^67,68^. The cleavage was conducted at room temperature for 2 hours. Then the supernatants were collected by filtration through a solid-phase synthesis tube with fritted disc of medium porosity, and concentrated using nitrogen flow. The crude product was obtained by precipitating the prior mixture into 50 mL of cold diethyl ether. After centrifugation, the pellets were dried under vacuum and re-dissolved in water containing 5% of acetic acid. The crude peptides were then purified by High-Pressure Liquid Chromatography (HPLC, 1260 Infinity, Agilent Technologies) equipped with a C8 reverse-phase column (300-5-C8, 21.2 × 100 mm, Kromasil). The HPLC gradient for purification is shown as follow: 0–3 minutes, from 10 to 15% ACN; 3–25 min, from 15 to 30% ACN; 25–30 min, from 30–100% ACN; 30 to 35 min, 100% ACN; 35–36 min, from 100 to 10% ACN. The flow rate was 6 mL/min. The purified peptides were isolated by lyophilization from HPLC elutes.

### Mass spectrometry

The molecular weight (MW) of synthesized peptides was measured by matrix-assisted laser desorption/ionization-time of flight (MALDI-TOF) mass spectrometry (MS) using a AXIMA Performance equipment (Shimadzu) at 45% of maximum laser power in the reflectron mode. The saturated solution of CHCA dissolved in a mixture of 49.95% H_2_O, 49.95% ACN, and 0.1% TFA was used as the matrix. To prepare the test samples, the matrix was mixed with the same volume of peptide sample (1 mg/mL) by vortexing. Then, 2 μL of the mixture was transferred onto the sample plate and left to dry at room temperature before the test.

### Coacervation studies

The peptides were resolubilized in 10 mM acetic acid buffer as stock solutions. To induce coacervation, the peptide stocks were mixed with various buffers whose pH ranged from 4 to 9 at a volume ratio of 1:9^18,19^. The receipts of buffers with different pH are listed in Supplementary Table 1. The ionic strength of buffers was fixed to 0.1 M and 1 M by adding salts including NaCl, NaBr, and Na_2_SO_4_. The formation of coacervates was observed by using inverted optical microscopy to determine the critical concentrations of GY23 variants required to undergo phase separation.

### SFA measurements

The adhesive property of peptide coacervates on mica surfaces was measured using an SFA 2000 (SurForce LLC, Santa Barbara)^69^. As described in previous studies^42,70^, freshly cleaved mica with a 55 nm silver layer deposited on its back was glued on the glass disks. Then 20 μL of freshly prepared peptide coacervates (5 mg/mL in the 50 mM Tris buffer with a pH of 8.0 and various IS fixed by different sodium salts) suspension was injected in the gap between disks with mica glued on their surfaces. The distance *D* between two mica surfaces was measured and calculated based on the fringes of equal chromatic order (FECO) technique. After the sample was injected into the gap, the system was equilibrated for 30 minutes by keeping two surfaces in contact with a bridging coacervate film. Then, the adhesion force of the peptide coacervates was measured by separating the two surfaces. The measured force F was normalized by the effective radius of the surface *R*. The interfacial energy *γ* conducted from the Young-Laplace equation of the coacervate phase/aqueous solution interface could be simplified as: $\gamma =F/4\pi R$^41^.

### Rheological studies

The rheological properties of peptide coacervates were measured by rheometry (MCR 501, Anton-Paar) with a cone-and-plate10 (CP10) geometry that has a fixed measuring gap of 47 μm. Considering the fast-aging nature of GY23 coacervates, traditional centrifugation is not able to achieve an ideal condense phase. Therefore, 70 μL of the condensed layer (5 mg/mL in the 50 mM Tris buffer with a pH of 8.0 and various IS fixed by different sodium salts), prepared by gently adding the peptide stock on top of the buffer without vigorously mixing^31^, was transferred to the rheometer to performe the test. Similar to previous studies^55^, strain sweep tests were performed from 0.1% to 10% of strain first for each sample at a constant frequency of 1 Hz to identify the linear viscoelastic (LVE) region. Then, frequency sweeps were conducted to measure the storage (G’) and loss (G”) moduli and the complex viscosity (*η*) of the coacervates at a constant 0.5% strain obtained from the LVE.

### Circular dichroism (CD) and Fourier-transform infrared (FTIR) spectroscopy

CD spectroscopy can provide information about the secondary structure of peptide coacervates. The prepared coacervate samples (5 mg/mL in 50 mM phosphate buffer with 8.0 of pH and 1 M of ionic strength fixed by KF) were added into a quartz sandwich cuvette with an optical path length of 0.2 mm. The signal was acquired by a CD spectrometer (Model 420, Aviv Biomedical Inc) with 1 nm resolution and 1 second of balancing time for each wavelength over 180 to 260 nm. Three scans were conducted for each sample. The collected spectra were averaged and smoothed at 12 pts by using Origin Pro 9.1 software as described by the literature^57^.

FTIR spectroscopy was also employed as a complementary method to identify the secondary structure of peptide coacervates. The spectra were collected at room temperature in the attenuated total reflection (ATR) mode on a FTIR spectrometer (Vertex 70, Bruker). Before the signal acquisition, 100 μL of coacervates suspensions were prepared by mixing 10 μL of stock solution (50 mg/mL in D_2_O containing 10 mM acetic acid) with 90 μL of buffers (50 mM phosphate D_2_O buffer with 8.0 of pH and 1 M of ionic strength fixed by KF), and then transferred to the surface of the ZnSe-diamond in the ATR accessory. The amide I band range from 1550 to 1720 cm^-1^ was collected and then processed to substrate water vapors and black sample, correct baselines, normalize signal intensities and deconvolute by OPUS 6.5 software^46,71^.

### Fluorescence recovery after photobleaching (FRAP)

To perform FRAP measurements, a fluorescence labeled peptide was prepared by adding a lysine residue (Lys) to the N-terminus of GY23 and GY23-9F/L, which was then reacted with FITC on the amine side chain of Lys. The labeled peptides, called KY24-FITC and KY24-9F/L-FITC, was mixed with pristine GY23 and GY23-9F/L at a ratio of 1/99 to prepare the stock solution with a final concentration of 50 mg/mL. The coacervates were prepared by pipetting the stock solution within the 50 mM Tris buffer (1:9 ratio) having a pH of 8.0 and an ionic strength (IS) of 1 M. The experiment started by applying a 488 nm laser pulse at the power of 30% on the chosen area of the coacervates from the confocal microscope (Eclipse Ti2, Nikon) to bleach the fluorescence. The confocal microscope then took images of the sample every 15 seconds. The fluorescence intensity was quantified by using ImageJ software and normalized by the intensity before the photobleaching.

### Supplementary information

Supplementary Information

Description of Additional Supplementary Files

Supplementary Data 1

## Data Availability

The authors declare that all relevant data supporting the findings of this study are available within the paper and its Supplementary Information file. All source data underlying the graphs and charts presented in the main figures are provided in the supplementary file “Supplementary Data 1”.
