# Supplementary material for: Tuning the viscoelastic properties of peptide coacervates by single amino acid mutations and salt kosmotropicity
Source: Commun Chem. 2024 Jan 4;7:5. doi: 10.1038/s42004-023-01094-y (PMC10766971; doi:10.1038/s42004-023-01094-y)
Supplement: Supplementary file 1 — Supplementary Information [file 42004_2023_1094_MOESM1_ESM.pdf]

Supplementary Information

Tuning the Viscoelastic Properties of Peptide Coacervates by Single Amino Acid Mutations and Salt Kosmotropicity

Xi Wu[1], Yue Sun[1], Jing Yu[2,3] *, Ali Miserez[1,4] *

[1]Biological and Biomimetic Material Laboratory (BBML), Center for Sustainable Materials (SusMat), School of Materials Science and Engineering, Nanyang Technological University, Singapore 637553.

[2]School of Materials Science and Engineering, Nanyang Technological University, Singapore 637553.

[3]Institute for Digital Molecular Analytics and Science, Nanyang Technological University, Singapore 637553.

[4]School of Biological Sciences, 60 Nanyang Drive, NTU, Singapore 636921.

*Author for correspondence: yujing@ntu.edu.sg, ali.miserez@ntu.edu.sg

Supplementary Table 1 The receipts of buffers used for coacervation study.

pH	Buffer salt
4	50 mM sodium acetate
5	50 mM sodium acetate
6	50 mM sodium bicarbonate
7	50 mM sodium dihydrogen phosphate
8	50 mM sodium dihydrogen phosphate
9	50 mM Tris·HCl

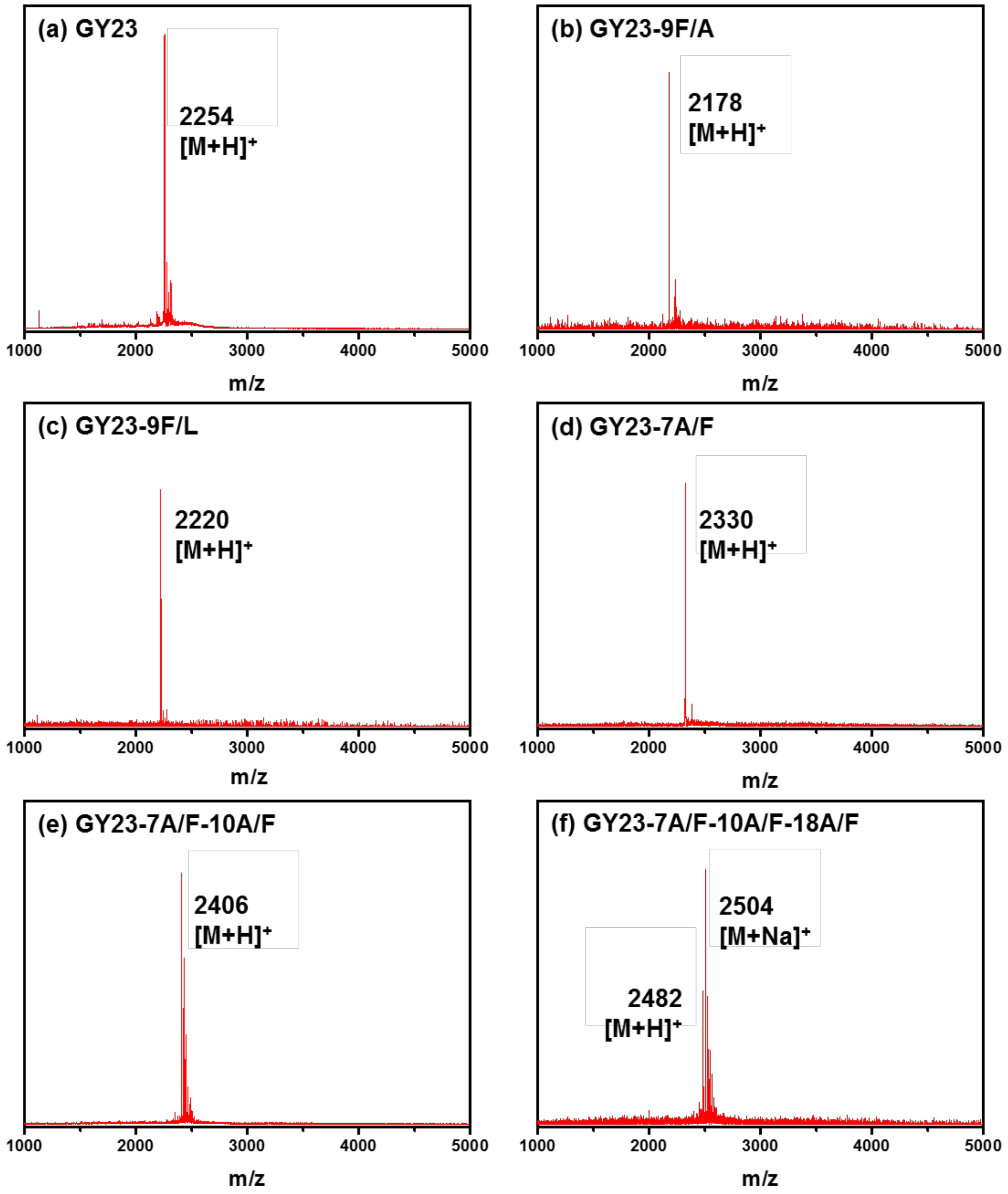

Supplementary Figure 1. MALDI-TOF spectra of GY23 variants.

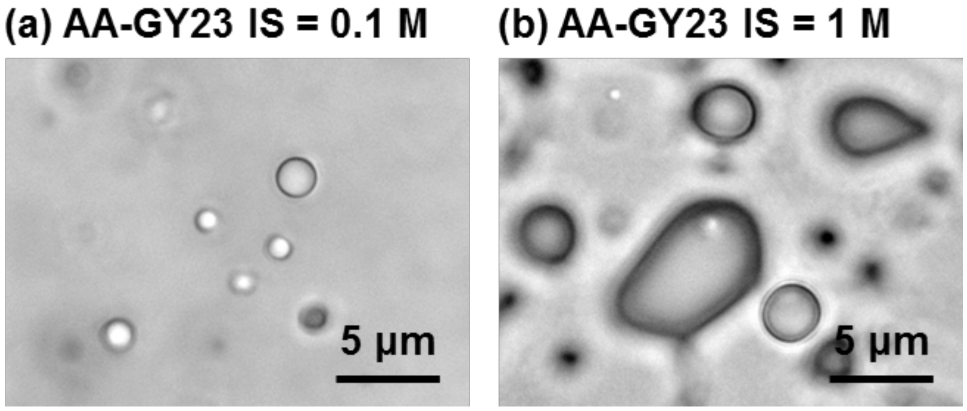

Supplementary Figure 2. Representative optical micrographs of AA-GY23 coacervates (5 mg/mL). Coacervates were prepared in pH 8.0 buffer with **(a)** 0.1 M and **(b)** 1 M of ionic strength fixed by NaCl.

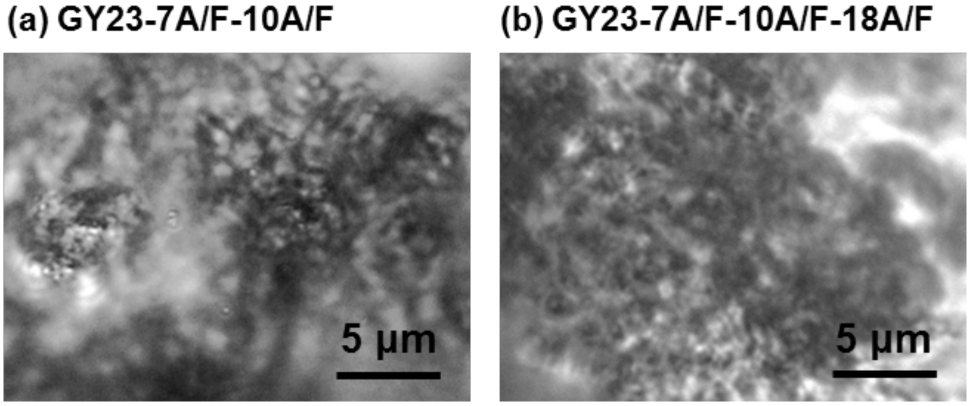

Supplementary Figure 3. Representative optical micrographs of GY23 variants with more A to F mutations. **(a)** GY23-7A/F-10A/F (5 mg/mL) and **(b)** GY23-7A/F-10A/F-18A/F (5 mg/mL) in pH 8.0 buffer with 1 M of ionic strength fixed by NaCl.

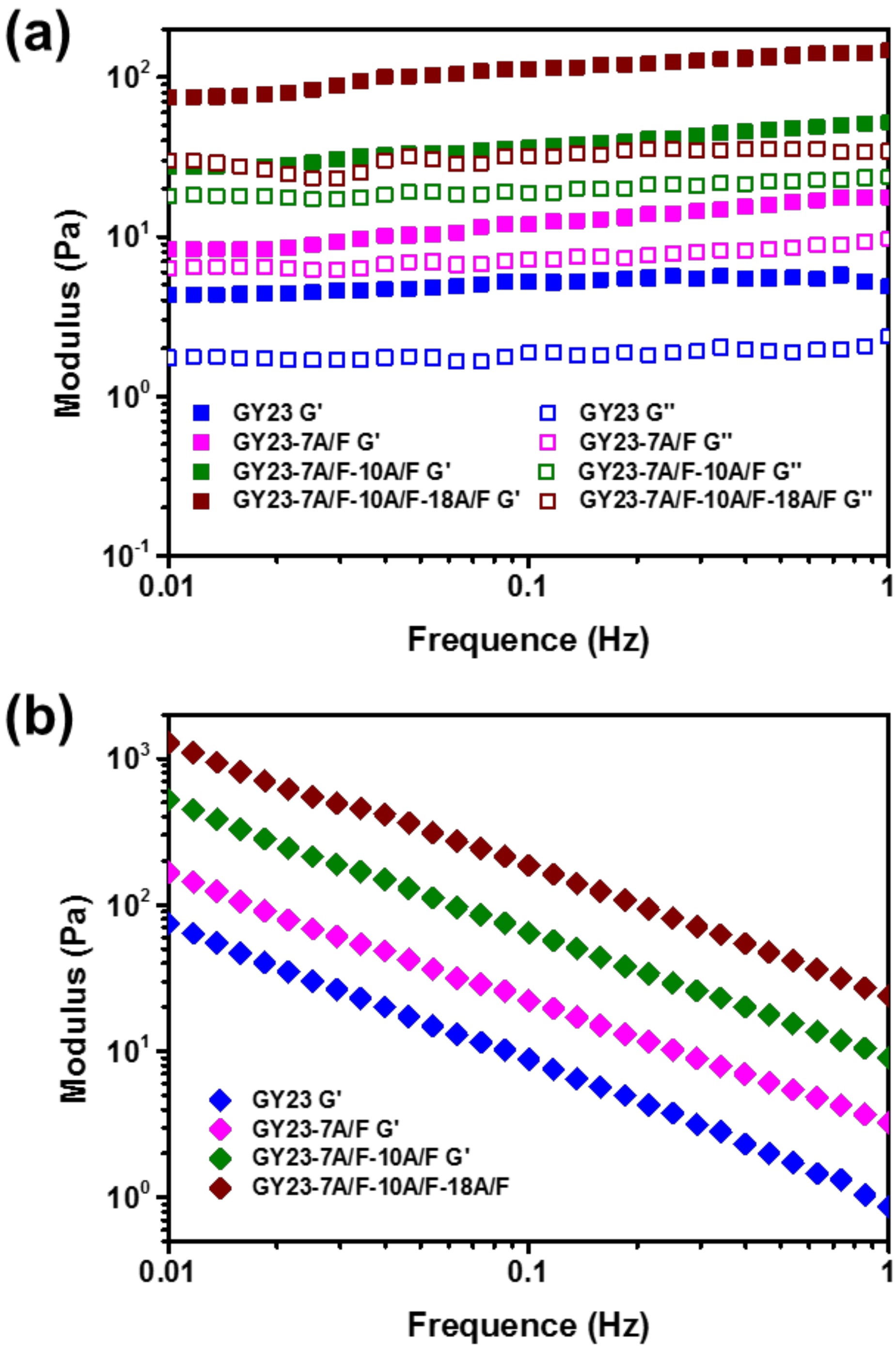

Supplementary Figure 4. Rheological properties of coacervates formed by GY23 variants with A to F mutations. **(a)** Storage modulus (*G*') and loss modulus (G'') of peptide coacervates. **(b)** Complex viscosity of peptide coacervates.
