# Supplementary material for: Tuning the viscoelastic properties of peptide coacervates by single amino acid mutations and salt kosmotropicity
Source: Commun Chem. 2024 Jan 4;7:5. doi: 10.1038/s42004-023-01094-y (PMC10766971; doi:10.1038/s42004-023-01094-y)
Supplement: Supplementary file 2 — Description of Additional Supplementary Files [file 42004_2023_1094_MOESM2_ESM.pdf]

Description of Additional Supplementary Files

File name: Supplementary Data 1

Description: Data for Figure 1, Figure 3, Figure 4, Figure 5, Figure 6
